# NRG‐1 Promotes Axon Regeneration of Dorsal Root Ganglion Neurons in Diabetic Rats via ITGB1/FAK/AKT Pathway

**DOI:** 10.1155/ijog/5520810

**Published:** 2025-09-23

**Authors:** Peng Zhan, Hewei Xiong, Hongmei Zheng

**Affiliations:** ^1^ Breast Cancer Center, Hubei Cancer Hospital, Tongji Medical College, Huazhong University of Science and Technology, National Key Clinical Specialty Discipline Construction Program, Hubei Provincial Clinical Research Center for Breast Cancer, Wuhan Clinical Research Center for Breast Cancer, Wuhan, Hubei, China, hust.edu.cn; ^2^ Department of Emergency Surgery, Union Hospital, Tongji Medical College, Huazhong University of Science and Technology, Wuhan, China, tjmu.edu.cn

**Keywords:** axons, ganglion cells, integrins, nerve regeneration, neuregulin (NRG), Type I diabetes

## Abstract

**Objectives:** The purpose of this study was to investigate the role of the ITGB1/FAK/AKT pathway in NRG‐1 in mediating the axonal growth of dorsal root ganglion neurons in Type I diabetic rats and to explore the mechanism of neuronal axonal regeneration.

**Result:** The effects of NRG‐1 on neurite outgrowth in diabetic rats were determined by protein quantification and axon length analysis in which axons were identified with tubulin‐*β*III‐specific staining. Western blot showed that the signaling activity of the ITGB1/FAK/AKT pathway was decreased in diabetic rats compared with normal rats, and exogenous NRG‐1 significantly promoted axonal growth and activated the pathway. After the addition of ITGB1‐specific siRNA, the axonal growth–promoting effect of NRG‐1 was partially reversed.

**Conclusion:** NRG‐1 promotes axonal outgrowth in diabetic DRGs via upregulating the ITGB1/FAK/AKT pathway, providing a potential target for diabetic neuropathy.

## 1. Introduction

Diabetic neuropathy is one of the common complications of chronic diabetes, which induces the peripheral processes of sensory neuron axons, including segmental demyelination or axon atrophy and loss. Deficient neurotrophin support has been proposed to contribute to the development of diabetic neuropathy, as the capacity to reorganize and regrow axons is compromised due to reduced neurotrophic support [1]. Endogenous neurotrophic factor, such as neuregulin (NRG), synthesized and released by central nervous system (CNS) neurons, promotes neuronal maturation and participates in brain development. Insufficient neurotrophic support aggravates sensory deficits in diabetes, owing to its function in maintaining the survival of specific neuronal populations [2]. The aim of this study was to explore the effect of NRG‐1 on mediating axonal growth in peripheral neurons of diabetic rats and its mechanism.

NRG‐1 comprises a group of polypeptide neurotrophic factors, with several subtypes (such as NRG‐1*α*, NRG‐1*β*, and NRG‐1*γ*), among which NRG‐1*β* is the most abundant in the dorsal root ganglia (DRGs). Through the NRG‐1/ErbB signaling pathway, it performs functions related to neuronal development and synaptic maintenance in the peripheral nervous system (PNS) [3–5]. Recent works have demonstrated that NRG‐*β*1 and its receptor subunits ErbB2, ErbB3, and ErbB4 are widely expressed in DRG neurons of rats [6, 7]. Moreover, NRG‐1*β* can act as an extracellular signaling factor to increase neuronal area and total neurite length and modulate the outgrowth of primary neurites in E18 hippocampal neurons via the p42/44 ERK and PKC signaling pathways [8].

Some researchers have detected that ITGB1 mRNA is differentially expressed in DRG tissues between streptozotocin (STZ)‐induced diabetic rats and normal control [9]. Interestingly, it has been reported that ErbB2 interacts with ITGB1 at the plasma membrane, which plays an important role in several cellular processes such as differentiation of various tissues as well as polarization and migration of epithelial cells [10–13]. Therefore, we hypothesized that the promoting effect of NRG‐1 on the growth of neuronal axons in diabetic rats may be related to its stimulation of ITGB1 and downstream pathways.

## 2. Method

### 2.1. Induction of Diabetes Mellitus (DM) Rats

All rats had free access to food and water under a 24‐h light‐dark cycle, and all experimental procedures were approved by and conducted in accordance with the guidelines of the Animal Care Committee of Tongji Medical College. Male Sprague–Dawley (SD) rats (weighing 180–220 g) were acquired and acclimatized for 1 week under standard laboratory conditions. The day before the induction of diabetes, the rats were fasted for 12 h but allowed free access to water. Diabetes was induced by a single intraperitoneal injection of STZ (75 mg/kg, Sigma‐Aldrich, United States) [14, 15]. Control rats received an equivalent volume of citrate buffer, adjusted for body weight. Blood glucose levels from the tail vein were monitored using a glucometer and test strips (ACCU‐CHEK Active, Basel, Switzerland). Rats with sustained blood glucose concentrations exceeding 16.8 mM for one consecutive week were included in the study.

### 2.2. Cell Isolation and Culture

As described previously in the Method section, cultures of DRG neurons were seeded at a density of 5 × 10^4^ cells per well in six‐well plates (coated with poly‐D‐lysine). This density was chosen to ensure minimal cell–cell contact while maintaining sufficient cell viability for axonal outgrowth assays [16]. Rats were sacrificed via decapitation under deep anesthesia induced by sodium pentobarbital. Bilateral DRGs from both the cervical and lumbar regions were collected from each rat. The tissue was dissociated in collagenase for 40 min and in trypsin for 20 min at 37°C. The cells are cultured in poly‐DL‐ornithine hydrobromide coated plates with Neurobasal medium supplemented with 2% B27 (Invitrogen, Carlsbad, CA), 2 mM L‐glutamine, 1% penicillin–streptomycin, and 50 ng/mL NGF at 37°C.

### 2.3. Western Blot

Western blotting was performed following our laboratory’s standard protocol [17]. Briefly, protein samples were extracted and lysed using RIPA buffer supplemented with 1 mM PMSF. The proteins were separated by SDS‐PAGE (Beyotime Biotechnology, China), transferred onto a PVDF membrane (Millipore, United States), and blocked with 5% nonfat dry milk in TBS. The membranes were then incubated overnight at 4°C with the following primary antibodies: anti‐tubulin‐*β*III (1:1000, CST, United States), anti‐AKT (1:1000, CST, United States), anti‐FAK (1:1000, CST, United Kingdom), anti‐p‐FAK (1:500, Abcam, United Kingdom), anti‐ITGB1 (1:1000, Proteintech, China), anti‐GAPDH (1:1000, Abcam, United Kingdom), anti‐GAP‐43 (1:3000, Abcam, United Kingdom), and anti‐Tau (1:1000, Abcam, United Kingdom). Subsequently, the membranes were incubated with horseradish peroxidase (HRP)–conjugated secondary antibodies for 2 h at room temperature. Protein bands were visualized using an ECL detection reagent and exposure to x‐ray film.

### 2.4. qRT‐PCR

Total RNA was extracted from DRGs using TRIzol reagent (TaKaRa, Dalian, China). cDNA was synthesized with the M‐MLV reverse transcriptase kit (ELK Biotechnology, EQ002). The qRT‐PCR was applied using EnTurbo SYBR Green PCR (qRT‐PCR) SuperMix kit (ELK Biotechnology, EQ001). The primer sequences used were as follows: ITGB, forward: ATGCTATCCCAACTACACTGGC, reverse: CACCAAGGCAGGTCTGACAG and *β*‐actin, forward: CGCTAACATCAAATGGGGTG, reverse: TTGCTGACAATCTTGAGGGAG. Expression of the targeted gene based on the threshold cycle was analyzed using the 2^−*Δ* *Δ*Ct^ method.

### 2.5. Immunostaining

The DRGs isolated from diabetic rats on 25 mm glass cover slips were fixed with 4% paraformaldehyde. Then, the primary antibody was added and incubated overnight at 4°C. For immunocytochemistry, rabbit anti‐tubulin‐*β*III (1: 200, CST, United States), which is neuron‐specific, is used. The coverslips were incubated with Alexa Fluor 488–conjugated secondary antibodies (1:300, CST, United States) for 1 h on the second day. Images were captured using a fluorescence microscope.

### 2.6. Axon Length Measurement

Confocal image stacks were flattened, and neurite branches were traced using a tracer plugin named Neuron J in FIJI (Fiji Is Just ImageJ). After setting the spatial scale (micrometers per pixel), the axonal length was calculated by multiplying the total number of pixels in the trace by the length corresponding to a single pixel. For dissociated cultures, the longest neurite of 20 DRGs selected randomly from independent optical fields in each group was measured.

### 2.7. Small Interfering RNA (siRNA) Silencing

Two integrin *β*1 siRNA was synthesized by RiboBio Co. Ltd. (Guangdong, China). ITGB1 target sequence of siRNA‐1 was GGACGAAAGTGCTCTAACA, and for siRNA‐2, it was CCAGATGGAGTAACAATAA. The confirmation of no obvious off‐target sequence of siRNA‐2 was supplemented by BLAST analysis. Briefly, the extracted diabetic DRGs were cultured in Neurobasal/B27 cultured medium (nonserum) before transfection. siRNA (20 nM) was diluted into riboFECT CP Buffer to make Solution A, which was subsequently added to Lipofectamine 2000 (Invitrogen, Carlsbad, CA). The cultured cells in six‐well plates were incubated with Solution A for 72 h, prior to conducting western blot and immunofluorescence analysis. Cells treated with nonspecific siRNA were used as the negative control.

### 2.8. Statistical Analysis

Data from more than two groups was analyzed by one‐way ANOVA with post hoc Dunnett’s test. Student’s *t*‐test was used to compare two groups. The data was expressed as mean ± SEM. Every experiment has at least three replicates independently. When *p* < 0.05, the data is of statistical significance on GraphPad Prism 7.

## 3. Result

### 3.1. STZ‐Induced Diabetes Rats

After 3 days of STZ (75 mg/kg) being administered via intraperitoneal injection, STZ was dissolved in 0.1 M citrate buffer (pH 4.5) freshly prepared on the day of injection.

Injections were performed within 30 min of dissolution to avoid degradation. The blood glucose of the DM group was significantly increased (> 16.8 mM) compared with the control group, and the symptoms of polydipsia and polyuria gradually appeared. Blood glucose was measured daily for the first week post‐STZ injection, then weekly for 8 weeks. After 8 weeks of feeding, three rats of the DM group died, and the average blood glucose concentration of the surviving rats is a 3.8‐fold increase compared to sham‐injected rats (25.7 ± 1.28 mM vs. 6.74 ± 0.64 mM, respectively). In terms of body weight, the DM rats decreased 36% compared to control rats (Table 1).

**Table 1:** Blood glucose levels and weight of control, DM group.

**Groups**		**Number**	**Blood glucose (mM)**	**P**_1_	**Weight (g)**	**P**_2_
Before induction	Control	10	6.31 ± 0.31	0.85	195.4 ± 3.578	0.2934
	DM	30	6.51 ± 0.19		190.3 ± 2.466	

4 weeks after induction	Control	10	7.31 ± 0.51	< 0.0001	273.6 ± 4.817	< 0.0001
	DM	28	26.25 ± 0.92		236.7 ± 3.208	

8 weeks after induction	Control	10	6.74 ± 0.64	< 0.0001	393.2 ± 9.459	< 0.0001
	DM	27	25.7 ± 1.28		251.4 ± 5.246	

*Note:* *P*_1_ means the blood glucose of the control group versus the DM group. *P*_2_ means the weight of the control group versus the DM group. Significant differences are analyzed by unpaired Student’s *t*‐test. Data are represented as mean values ± SEM.

### 3.2. ITGB1/FAK/AKT Pathway Has a Low Expression in Diabetic Rat DRGs

ITGB1 is an important participating molecule in the process of axon regeneration, and whether it is differently expressed in DRG neurons of diabetic rats remains unknown.

We isolated sensory neurons as described in the Method section. Adult DRGs from control and STZ‐induced diabetic rats were cultured for 72 h under defined conditions. RT‐qPCR results showed that the mRNA level of ITGB1 was decreased by 40% (*p* < 0.05) in the DM group compared with the NC group (Figure 1a). Western blot showed that the activity of the ITGB1/FAK/AKT pathway was decreased in the DM group (Figure 1b). The difference was statistically significant (Figure 1c). Therefore, these data prompted us to explore the role of the ITGB1/FAK/AKT pathway in DRGs of diabetic rats.

Figure 1Expression of ITGB1 in diabetic rats. (a) RT‐qPCR result of ITGB1 mRNA in DRG neurons from normal (*n* = 3) and diabetic rats (*n* = 3). (b) Western blot analysis of ITGB/FAK/AKT pathway protein expression from these two groups. (c) Quantification of proteins using ImageJ. Data was reported as mean ± SD.  ^∗^ *p* < 0.05 and  ^∗∗^ *p* < 0.01 versus NC group. NC, negative control; DM, diabetes mellitus; ITGB1, integrin‐beta1; FAK, focal adhesion kinase; DRG, dorsal root ganglion.

**Figure:**
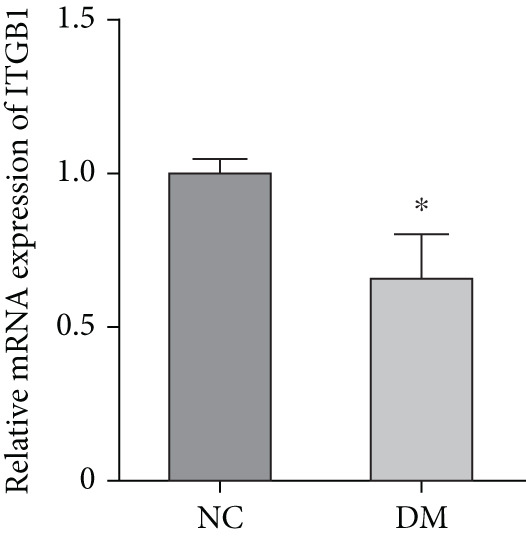
(a)

**Figure:**
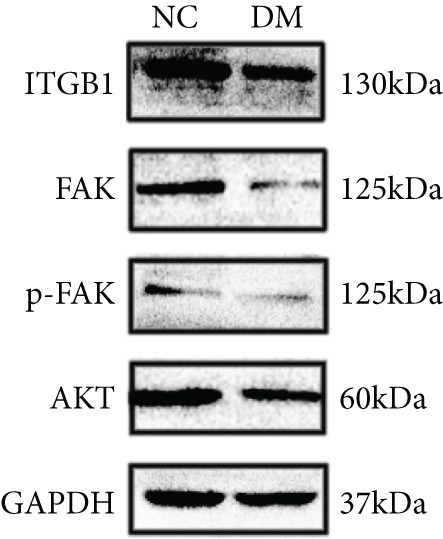
(b)

**Figure:**
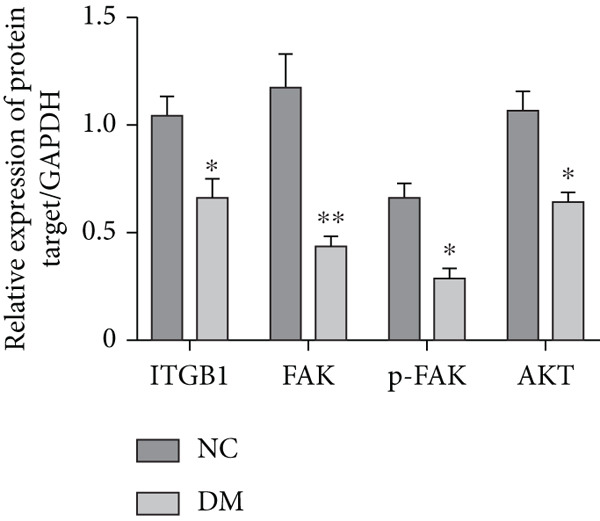
(c)

### 3.3. Effects of Different NRG‐1 Concentrations on Axon Regeneration of DRGs

The suitable concentration of NRG‐1 promoting neuronal axon regeneration from diabetic rats needs to be explored. Immunofluorescence images were used to analyze the length of axon regeneration, and protein expression of Tau and GAP‐43 was analyzed by western blot to reflect the molecular level of axon regeneration.

The level of GAP‐43 protein expression is closely linked to nerve regeneration, which exerts a key effect on recovering functional and structural plasticity of axon for reconstruction [18–20]. It has been reported that Tau expression at both the protein and mRNA levels is upregulated in adult rat DRG regenerating neurons [21].

Immunofluorescence and grayscale value analysis showed that all treated groups promoted axon growth to different extents (Figure 2c) and upregulated GAP‐43 and Tau expression (*p* < 0.05) (Figure 2a,b). However, the axon regeneration length of neurite was longest at 20 ng/mL (Figure 2c), and the difference compared to the 10 or the 50 ng/mL group (*p* < 0.05) was statistically significant (Figure 2d). Therefore, 20 ng/mL was considered the optimal concentration in subsequent experiments because of its most pronounced promotive effect.

Figure 2DRGs from diabetic rats treated with different concentrations of NRG‐1 for 72 h. (a) Western blot result of GAP‐43 and Tau expression in cells of five groups and (b) band analysis using ImageJ software. (c) Immunofluorescence staining of tubulin‐*β*III in cells of each group, scale bar 50 *μ*m. (d) ImageJ software used to analyze neurite length (micrometers).  ^∗^ *p* < 0.05,  ^∗∗^ *p* < 0.01, and  ^∗∗∗^ *p* < 0.001 versus NC group. ^#^ *p* < 0.05, ^##^ *p* < 0.01, and ^###^ *p* < 0.001 versus 20 ng/mL group. NRG, neuregulin; GAP‐43, growth‐associated protein; DRGs, dorsal root ganglion neurons.

**Figure:**
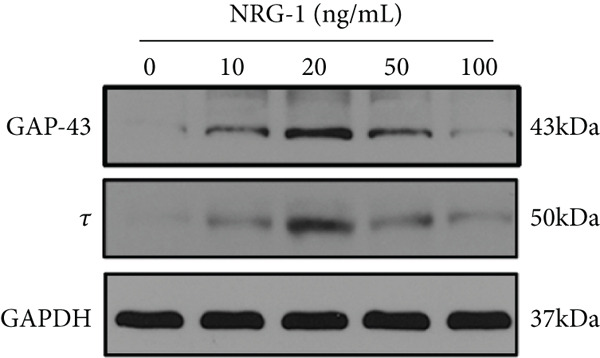
(a)

**Figure:**
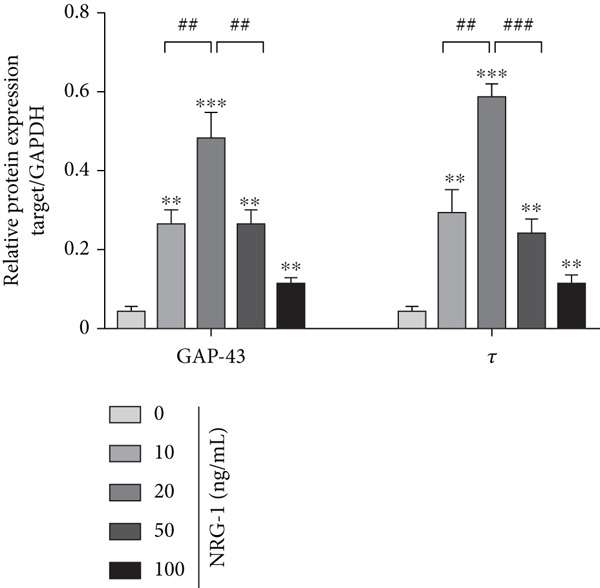
(b)

**Figure:**
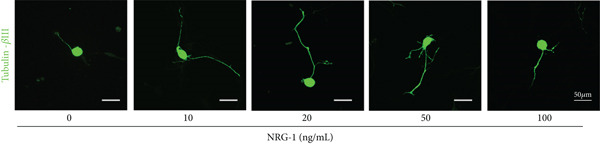
(c)

**Figure:**
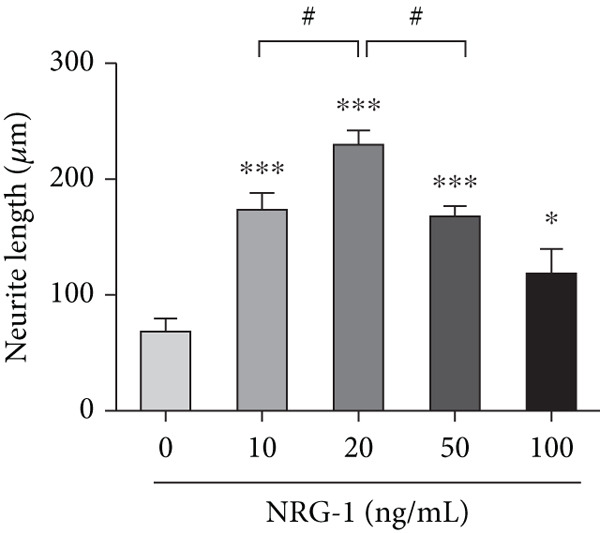
(d)

### 3.4. siRNA Effectiveness Test

DRGs from diabetic rats were treated with two specific ITGB1‐siRNAs and a control siRNA. After 72 h of transfection, the silencing effect was analyzed by western blot. The expression of ITGB1 in the siRNA‐2 group was significantly decreased (Figure 3a). The difference between this group and the negative control group or siRNA‐1 group or transfection reagent control group was statistically significant (*p* < 0.05) (Figure 3b). Thus, in the subsequent experiments, we selected siRNA‐2 as the interfering RNA.

Figure 3siRNA interference effect. (a) Western blot analysis of ITGB1 protein in sensory neurons. (b) Relative gray value analyses were conducted.  ^∗∗^ *p* < 0.01 and  ^∗∗∗^ *p* < 0.0001 versus NC group. ^#^ *p* < 0.05 versus siRNA‐2 group. siRNA, small interference RNA.

**Figure:**
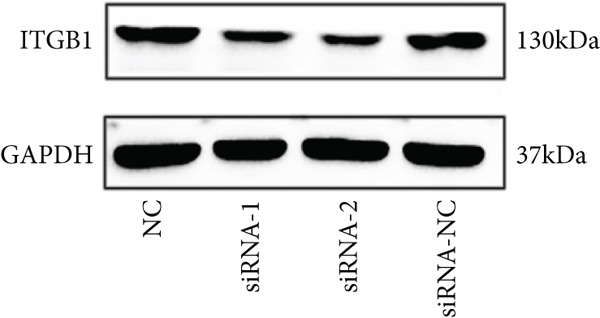
(a)

**Figure:**
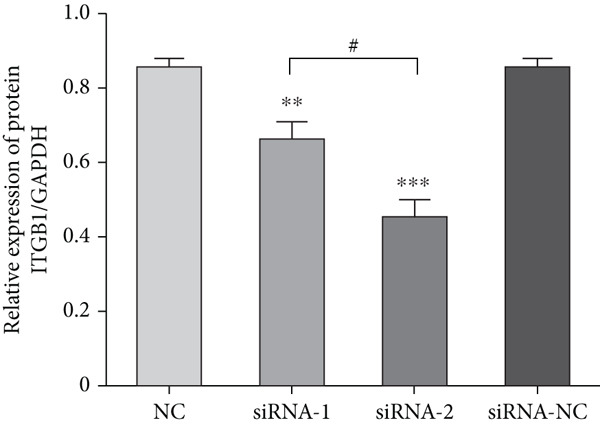
(b)

### 3.5. NRG‐1 Promotes Axon Outgrowth by Enhancing ITGB1/FAK/AKT Pathway Expression

**Figure 4:**
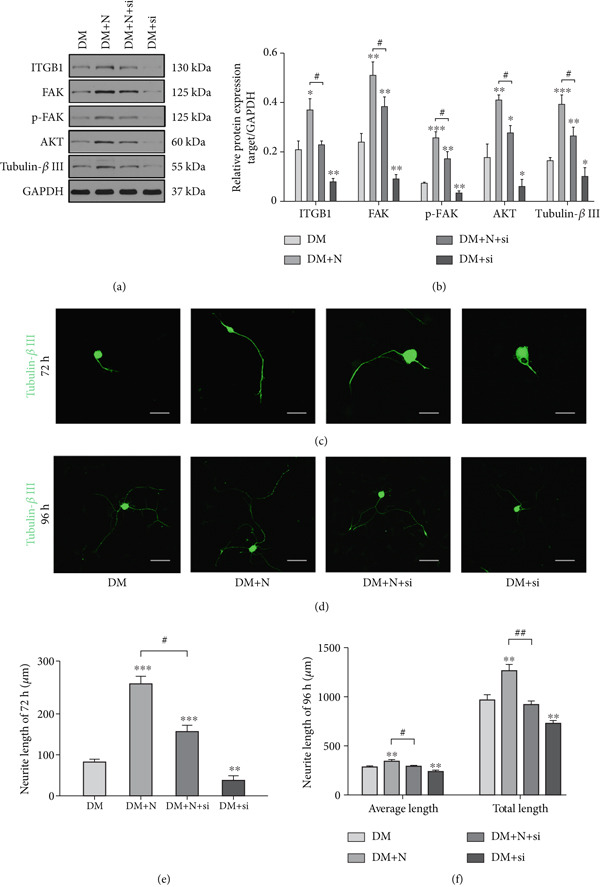
NRG‐1 promotes axonal outgrowth via ITGB1/FAK/AKT pathway. (a) Western blot analysis of ITBA1, FAK, p‐FAK, AKT, tubulin‐*β*III from diabetic rat DRGs after being treated with NRG‐1 and/or siRNA for 72 h. (b) Quantification of protein using ImageJ. Immunofluorescence staining of tubulin‐*β*III in (c) 72 h (scale bar 50 *μ*m) and (d) 96 h (scale bar 100 *μ*m). (e, f) ImageJ software analyzes neurite length of (c) and (d) (micrometers).  ^∗^ *p* < 0.05,  ^∗∗^ *p* < 0.01, and  ^∗∗∗^ *p* < 0.001 versus NC group. ^#^ *p* < 0.05, ^##^ *p* < 0.01, and  ^∗∗∗^ *p* < 0.001 versus DM+N group.

To explore the relevance of the ITGB1/FAK/AKT pathway to NRG‐1 stimulated axonal regeneration, DRGs were transfected with siRNA and then analyzed by western blot and immunofluorescence. At 72 h, the results showed that the neurite length of the DM+N group increased by 3.33‐fold (Figure 4c) compared with the DM group (250.5 ± 9.306 * μ*m vs. 75.04 ± 2.929 * μ*m, respectively) and simultaneously enhanced the expression of the ITGB1/FAK/AKT pathway and tubulin‐*β*III (Figure 4a,b). However, when siRNA was added, this promotion of axonal regeneration was greatly reversed by approximately 40% (143.5 ± 6.15 * μ*m vs. 250.5 ± 9.306 * μ*m, respectively), accompanied by downregulation of this pathway and tubulin‐*β*III. Compared with the DM group, the DM+si group significantly inhibited the growth of axons.

When we cultured neuronal cells for 96 h, the immunofluorescence results showed that the axons in each group exhibited increased density and significantly greater length (Figures 4d, 4e, and 4f). The average and total neurite length of the DM+N group increased by 20% compared to the DM group. Surprisingly, there was no statistically significant difference in average and total neurite length between the DM group and the DM+N+si group.

These results indicate that exogenous NRG‐1 increases the axonal regeneration ability of diabetic rats DRGs, likely by promoting nerve repair through modulation of ITGB1/FAK/AKT expression. Silencing ITGB1 can reverse this effect and reduce the promotive effects.

## 4. Discussion

Nerve fiber loss was found during diabetic peripheral neuropathy, leading to dysesthesia which might put patients at a high risk of foot ulceration and serious injuries [22]. Previous research demonstrates that sural nerve fibers of patients with DNP analyzed by nerve biopsies are fewer than those with diabetes but no DPN [23]. Furthermore, some pathological changes of endoneurial microvascular, such as gross basement membrane thickening, endothelial cell proliferation, and hypertrophy, are more severe in DNP patients, which means more serious segmental demyelination or axon atrophy and loss [24].

Integrins, a family of heterodimeric transmembrane and cell adhesion receptors, mainly recognize extracellular matrix (ECM) ligands and cell surface ligands [25]. They consist of *α* and *β* subunits, among which integrin *β*1 is a highly distributed subunit [26]. Integrin *β*1 has been validated to be expressed in sensory neurons of the DRG of rats and stimulates axonal outgrowth in response to the ECM, an effect that could be blocked by using subunit‐specific antibodies [27]. The upregulation of mRNAs for integrin subunits is detected as the corresponding proteins in regenerating axons. This could be considered a response mechanism important for neurite regeneration after sciatic nerve lesions [28]. In this study, we detected a decrease in the expression of the ITGB1/FAK/AKT pathway in DRGs of diabetic rats compared to normal rats, which may provide a breakthrough in the study of demyelination or axonal atrophy in diabetic neuropathy. The STZ‐induced mortality observed is justified by the establishment of a severe diabetic state, essential for modeling neuropathy. The chosen dose (75 mg/kg) reliably produced a valid diabetic phenotype without compromising the statistical power of the study.

The fact that NRG‐1b can stimulate neurite extension via the ErbB4 receptor–p42/44 MAPK signaling pathways in embryonic hippocampal neurons is well known [8]. It is possible that NRG‐1 can promote the growth of axons through other pathways. As is known, integrin *β*1/FAK/AKT signaling has a pivotal role in the metastatic ability of cancer cells [29]. Recent reports have shown that the upregulation of FAK and integrin *β*1 phosphorylation expression is detected in the spinal cord after rats suffer chronic constriction injury [30]. Our experiment demonstrates that exogenous NRG‐1 upregulates ITGB1/FAK/AKT pathway expression in DRGs of diabetic rats and accelerates the regeneration of neuronal axons.

We used GAP‐43 and Tau proteins to assess changes at the molecular levels during axonal regeneration after treatment with different concentrations of NRG‐1.

The level of GAP‐43 protein expression is closely linked to nerve regeneration, which exerts a key effect on recovering functional and structural plasticity of axons for reconstruction [18–20]. Researchers have reported that Tau expression at both the protein and mRNA levels is upregulated in adult rat DRG regenerating neurons [21].

GAP‐43 is discovered as an intracellular growth–associated protein, which exerts an important function in stimulating the transduction of signals to mediate cytoskeletal organization at the end of the neurite [31–33]. The process of neuronal growth is accompanied by this regulation of GAP‐43, and these processes may continue in mature animals [34]. GAP‐43 is mainly used in developmental and in vitro studies to evaluate axonal growth. Tau is a natively unfolded microtubule‐binding protein which is found expressed abundantly in the axon of neuronal cells and plays an important role in the assembly and stabilization of microtubules.

When we analyzed the reinforced regeneration ability of axon by NRG‐1, the result conformed to the findings of previous research, showing NRG‐1 exerts differential promotive effects on axonal regeneration compared to NGF. NRG‐1 has more potential to promote neurite elongation, while NGF has more advantages in promoting neurite density and branching [35, 36].

In conclusion, our results indicate that ITGB1 mRNA expression is attenuated in diabetic rats and that the expression of the ITGB1/FAK/AKT pathway is decreased. However, under the stimulation of exogenous NRG, DRGs exhibited the ability to enhance axonal growth and upregulated this pathway. Furthermore, when ITGB1‐siRNA was added, the expression of this pathway was further inhibited, partially reversing the promotion of axonal growth by NRG‐1. The results of this experiment may provide a breakthrough for the treatment of diabetic neuropathy.

## Ethics Statement

All animal experiments were conducted according to the guidelines and standards of the Experimental Animal Center of Tongji Medical College, Huazhong University of Science and Technology.

## Consent

The authors have nothing to report.

## Conflicts of Interest

The authors declare no conflicts of interest.

## Author Contributions

P.Z. and H.X. designed the experiments, and P.Z. analyzed the data. P.Z. wrote the paper, and the manuscript was reviewed by H.Z.

## Funding

This study was funded by the Hubei Province Medical Youth Top Talent Project (EWT[2023]65, Oncology), the Hubei Provincial Natural Science Foundation Innovation and Development Joint Fund Project (2024AFD454), the Chen Xiao‐Ping Foundation for the Development of Science and Technology of Hubei Province (CXPJJH123001‐2320), and the Hubei Cancer Hospital Biomedical Center Research Project (2022SWZX03 and 2022SWZX09).

## Data Availability

The experimental data included in this study can be obtained by contacting the corresponding author.
